# Opening up new fronts in the fight against cholesterol

**DOI:** 10.7554/eLife.00663

**Published:** 2013-04-09

**Authors:** Russell A DeBose-Boyd, Jay D Horton

**Affiliations:** 1 **Russell A DeBose-Boyd** is at the Department of Molecular Genetics and the Howard Hughes Medical Institute, University of Texas Southwestern Medical Center, Dallas, United StatesRussell.Debose-Boyd@utsouthwestern.edu; 2 **Jay D Horton** is at the Departments of Internal Medicine and Molecular Genetics, University of Texas Southwestern Medical Center, Dallas, United StatesJay.Horton@utsouthwestern.edu

**Keywords:** Secretory pathway, COP II, Cholesterol metabolism, Mouse

## Abstract

An unexpected connection between a secretory protein called PCSK9 and Sec24A, a well known protein-transport protein, could lead to the development of novel treatments for patients with high levels of low-density lipoproteins in their blood.

**Related research article** Chen X-W, Wang H, Bajaj K, Zhang P, Meng Z-X, Ma D, Bai Y, Liu H-H, Adams E, Baines A, Yu G, Sartor MA, Zhang B, Yi Z, Lin J, Young SG, Schekman R, Ginsburg D. 2013. SEC24A deficiency lowers plasma cholesterol through reduced PCSK9 secretion. *eLife* **2**:e00444. doi: 10.7554/eLife.00444

**Image** Mice with a deficiency of Sec24A are normal in many ways, as these liver cells show, and also have lower than normal levels of cholesterol in their blood

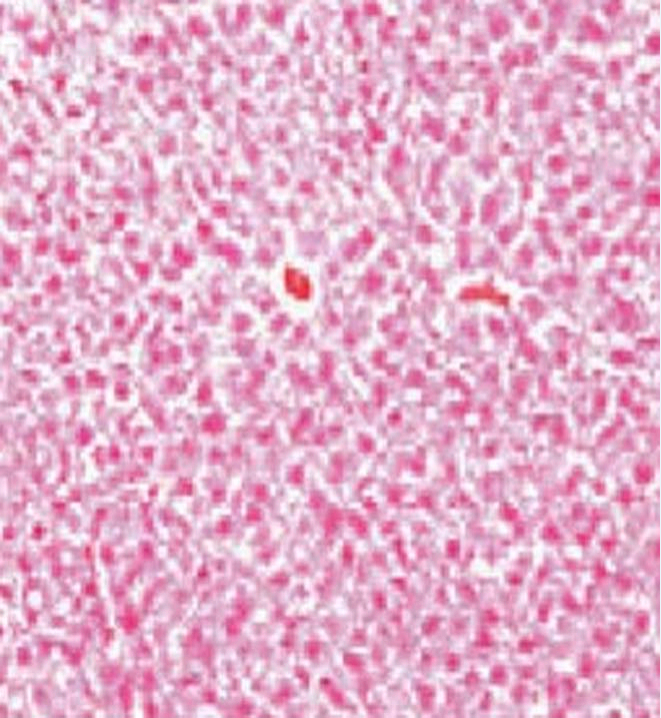

Since its discovery in 2003, the secretory protein PCSK9 has been the subject of growing interest because of its role in the degradation of low-density lipoprotein (LDL) receptors in the liver (Seidah et al., 2003). LDL receptors, which reside on the surface of liver cells, help to control cholesterol levels in the body by binding to low-density lipoprotein particles circulating in the blood and mediating their entry into the cells (Horton et al., 2009). Once inside the cells, the particles are released inside endosomes and are subsequently degraded in lysosomes, while the LDL receptors return to the cell surface to capture more low-density lipoprotein particles. PCSK9—which is short for proprotein convertase subtilisin-like type 9—prevents the LDL receptors from doing this job by binding to them and then diverting them to lysosomes for degradation. The physiological relevance of this reaction was first revealed by genetic analyses of humans with abnormal levels of lipids in their blood (Abifadel et al., 2003; Cohen et al., 2006), and confirmed in experiments using genetically manipulated mice (Maxwell and Breslow, 2004; Rashid et al., 2005; Lagace et al., 2006).

Gain-of-function mutations in PCSK9 lead to high levels of low-density lipoproteins in the blood (hypercholesterolemia) because they promote the degradation of LDL receptors, whereas loss-of-function mutations markedly reduce the levels of low-density lipoproteins. Since high levels of these lipoproteins are an important risk factor for atherosclerosis and associated coronary heart disease, finding ways to inhibit PCSK9 has become the focus of much research. Indeed, the results of recent clinical trials show that antibody-mediated inhibition of PCSK9 can reduce the levels of low-density lipoproteins in patients with hypercholesterolemia by as much as 65–70% (King, 2013). Now, in *eLife*, David Ginsburg of the University of Michigan and co-workers—including Xiao-Wei Chen as first author—report important insights into the transport of PCSK9 within cells (Chen et al., 2013).

PCSK9 is synthesized as a precursor in the endoplasmic reticulum (ER; Figure 1) and, like many other secretory proteins, it is subjected to post-translational modifications including glycosylation, phosphorylation, and tyrosine sulfation. Most studies to date have focused on PCSK9 once it has been secreted from cells. However, Chen and co-workers—who are based at Michigan, UC Berkeley, Wayne State University, the Cleveland Clinic and UCLA—have focused their attention on the secretion of PCSK9, and made the surprising discovery that decreased secretion of PCSK9 results in higher levels of LDL receptors, with a protein called Sec24A having a central role in the connection between the two.

**Figure 1.**
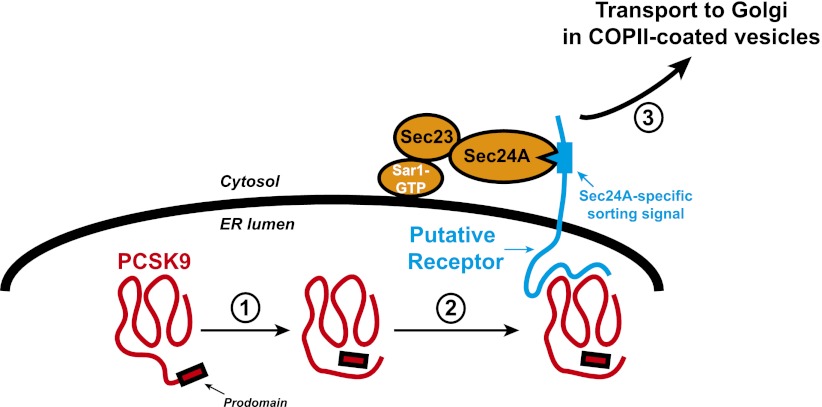
Transport of the secretory protein PCSK9 from the endoplasmic reticulum (ER) to the Golgi. When newly synthesized PCSK9 (shown in red) reaches the lumen of the endoplasmic reticulum (ER), it undergoes autocatalytic cleavage (1): This creates a prodomain that remains associated with the PCSK9 as it is exported from the ER and transported to the Golgi. Data from Chen et al. indicate that PCSK9 associates with a putative transmembrane receptor (shown in blue) that links it to a Sec23/Sec24A complex in the cytosol (2). This link is likely mediated by specific interactions between one or more sorting signals in the cytosolic domain of the receptor and binding sites in Sec24A. The receptor (along with PCSK9) is then incorporated into COPII-coated vesicles (not shown) for transport to the Golgi (3) and subsequent secretion. The Sar1 enzyme that triggers the formation of the vesicle, and its release from the ER membrane (thick black line), is also shown.

Proteins are transported from the ER to the Golgi by vesicles coated with coat protein complex II (COPII), which was first identified in yeast and consists of the enzyme Sar1, and complexes made of Sec proteins (notably the heterodimeric Sec23/Sec24 complex, and the heterotetrameric Sec13/Sec31). Sec24 mediates the packaging of the protein to be transported (which is called the cargo protein) into COPII-coated vesicles, and its importance is highlighted by the observation that deletion of the *Sec24* gene in yeast cells results in their death. Mammals express four paralogs or versions of Sec24—Sec24A, Sec24B, Sec24C and Sec24D—and the deletion of these paralogs in mice results in phenotypes that range from a severe neural closure defect when Sec24B is deleted, to early embryonic lethality when Sec24D is deleted (Zanetti et al., 2012). It seems likely, therefore, that each paralog mediates the inclusion of specific cargo proteins into vesicles for subsequent export from the ER.

The cargo proteins transported by COPII-coated vesicles include transmembrane proteins that span the ER membrane one or more times, as well as soluble proteins that are contained entirely within the lumen of the ER. Biochemical and crystallographic studies indicate that incorporation of a particular transmembrane cargo protein into the transport vesicle is mediated by a binding site on the Sec24 protein, which is in the cytosol, and a specific signal presented by the cargo protein (Mancias and Goldberg, 2008). How soluble proteins became incorporated into transport vesicles is not completely understood. According to the ‘bulk flow’ model, all soluble proteins become incorporated into COPII vesicles by default without selection. However, incorporation of some soluble proteins into transport vesicles is mediated by their binding to transmembrane receptors that present specific sorting signals to the Sec24 proteins in the cytosol (Figure 1).

The results of Chen and co-workers indicate that the latter of these two scenarios applies to the export of PCSK9 from the ER. They find that mice deficient in Sec24A are remarkably normal in terms of survival, development and fertility. However, characterization of these mice also led to an unexpected discovery: a deficiency of Sec24A causes abnormally low levels of low-density lipoproteins in the blood (hypocholesterolemia) as a result of elevated levels of LDL receptors in the liver.

At least three lines of evidence indicate that these high levels of LDL receptors result from decreased secretion of PCSK9. First, the levels of PCSK9 in plasma of Sec24A-deficient mice are reduced compared to wild type animals. This is accompanied by increased levels of PCSK9 within liver cells and increased expression of LDL receptors on the surface of liver cells. Second, Sec24A binds to PCSK9, even though Sec24A is a cytosolic protein and PCSK9 is confined within the lumen of the ER (Figure 1). Third, overexpression of Sec24 promotes secretion of PCSK9, whereas reducing Sec24A expression by RNA interference-mediated knockdown blunts packaging of PCSK9 into COPII vesicles.

The action of Sec24A appears to be restricted to a subset of proteins that includes PCSK9. The activation of membrane-bound transcription factors called SREBPs requires their transport from the ER to the Golgi to be mediated by the ‘escort’ protein Scap (Brown and Goldstein, 2009). Chen et al. find that SREBP activation (and thus its Scap-mediated transport from the ER to the Golgi), continues normally in the livers of Sec24A deficient mice. This is consistent with the finding that Sec24C mediates the incorporation of the Scap protein into COPII vesicles (Sun et al., 2007).

Despite these observations, enthusiasm for strategies that reduce the secretion of PCSK9 by inhibiting Sec24A should be tempered until other proteins that require Sec24A for secretion are identified. The work of Chen et al. suggests that it might be better to inhibit the putative receptor that links PCSK9 in the ER to Sec24A in the cytosol (Figure 1). Identifying this receptor and elucidating how it works will be important for two reasons: it will teach us more about the export of PCSK9 and other soluble proteins from the ER in mammals, and it might lead to the development of novel therapies to reduce the levels of low-density lipoproteins in the blood and therefore help prevent atherosclerosis and heart disease.
